# Spectral Clustering Algorithm for Cognitive Diagnostic Assessment

**DOI:** 10.3389/fpsyg.2020.00944

**Published:** 2020-05-15

**Authors:** Lei Guo, Jing Yang, Naiqing Song

**Affiliations:** ^1^Faculty of Psychology, Southwest University, Chongqing, China; ^2^Southwest University Branch, Collaborative Innovation Center of Assessment Toward Basic Education Quality, Chongqing, China; ^3^School of Mathematics and Statistics, Northeast Normal University, Changchun, China; ^4^Basic Education Research Center, Southwest University, Chongqing, China; ^5^Urban and Rural Education Research Center, Southwest University, Chongqing, China

**Keywords:** cognitive diagnostic assessment, spectral clustering, K-means, G-DINA model, classification accuracy

## Abstract

In cognitive diagnostic assessment (CDA), clustering analysis is an efficient approach to classify examinees into attribute-homogeneous groups. Many researchers have proposed different methods, such as the nonparametric method with Hamming distance, K-means method, and hierarchical agglomerative cluster analysis, to achieve the classification goal. In this paper, according to their responses, we introduce a spectral clustering algorithm (SCA) to cluster examinees. Simulation studies are used to compare the classification accuracy of the SCA, K-means algorithm, G-DINA model and its related reduced cognitive diagnostic models. A real data analysis is also conducted to evaluate the feasibility of the SCA. Some research directions are discussed in the final section.

## Introduction

In the past decades, there has been a significant increasing interest in cognitive diagnostic assessment (CDA) that allows for the purpose of identifying the presence or absence of specific fine-grained attributes required for solving problems on a test in educational and psychological assessment. Researchers have proposed a variety of methods to classify examinees into several categories by matching their attribute profiles. To sum up, there have been two major kinds of approaches till now. One of them usually uses cognitive diagnosis models (CDMs) to estimate the attribute profile for each examinee, which can be called parametric technique. The differences between these CDMs are assumptions about how cognitive attributes affect examinees’ responses in CDAs. The deterministic input; noisy “and” gate (DINA; Junker and Sijtsma, 2001), and noisy input; deterministic “and” gate model (NIDA; Junker and Sijtsma, 2001) are the typical conjunctive models, which require examinees must master all required attributes, thus even lacking one required attribute will lead to a totally wrong response. Disjunctive models, such as the deterministic input; noisy “or” gate model (DINO; Templin and Henson, 2006), suppose that if one has mastered a subset of required attributes, even merely one, the probability of a correct response will be sufficiently high. Other specific, interpretable CDMs include the linear logistic model (LLM; Maris, 1999) the additive CDM (A-CDM; de la Torre, 2011) and the reduced reparameterized unified model (RRUM; Hartz, 2002). To subsume the above reduced models, some general CDM frameworks has been proposed, such as the log-linear CDM (LCDM; Henson et al., 2009) the generalized DINA (G-DINA; de la Torre, 2011) model and the general diagnostic model (GDM; von Davier, 2008). The major advantage of general CDMs is that they have the largest flexibility of fetting response data which is set under the CDM framework, and it always should be taken into account at first when doing parameter estimation.

The superiority of parametric models is conciseness. However, one big issue inherently exists in the parametric technique, i.e. sample size. Several researchers have investigated the influence of sample size on estimation accuracy of the model parameters and pattern/attribute correct classification rate (de la Torre et al., 2010; Chen and de la Torre, 2013; Minchen et al., 2017). Although the results represented that sample size had a negligible impact on correct classification rate, most previous studies obtained this conclusion by setting the number of examinees no less than 500. So, there is no evidence to draw the inference that no effect on correct classification rate when using small sample size (may be less than 50 or 100). Virtually, the number of examinees in one class is not large for the most part. It is doubtful whether the performance of the parametric models is good or not when teachers implement the cognitive diagnostic test in class with a smaller sample size.

To address this issue, nonparametric techniques can be treated as alternative approaches to classify examinees into attribute-homogeneous groups, which is less restrictive and often computationally more efficient. Better yet, many nonparametric classification algorithms can be easily implemented in most statistical software packages. Based on the advantages of nonparametric techniques, many different methods have been proposed in the CDA. For example, three different methods of computing sum-scores (simple sum-scores, complex sum-scores, and weighted complex sum-scores) combined with model-based mastery sum-score cutoffs were proposed (Henson et al., 2007). Their results indicated that the correct classification rates of examinees’ attribute profiles from model-based sum-scores and mastery sum-score cutoffs were able to compare with those correct classification rates from CDM. Chiu et al. (2009) used hierarchical agglomerative clustering and K-means methods to group examinees into different clusters possessing the same attribute profiles. Simulation results demonstrated that K-means method had better performance at the classification consistency and homogeneity of a cluster than that of hierarchical agglomerative clustering in most experimental conditions. Subsequently, Chiu and Douglas (2013) proposed a nonparametric procedure that merely relied on a given Q-matrix (Tatsuoka, 1985), and evaluated the examinees’ attribute profiles by minimizing the distance measures (hamming distance, weighted hamming distance, and penalized hamming distance) between observed responses and the expected responses of a given attribute profile. Specifically, this procedure based on expected response patterns makes no direct use of item parameters of any CDMs. So, it required no parameter estimation, and can be used on a sample size as small as 1 (recall that the sample size is no less than 500 in CDMs based on existing studies). In addition, the existing studies have provided plenty of evidence that the nonparametric classification algorithms have good performance in CDA.

The primary objective of this paper is to introduce the method for implementing CDA using spectral clustering algorithm (SCA), which has become one of the most prevalent modern clustering methods in recent years. The SCA creates a graph of objects that require classifying based on the similarity measurement of each pair of objects (i.e. examinees in this paper). The more similar the examinees’ attribute profiles are, the greater probability they can interrelate with each other in the graph. Next, the examinees’ attribute profiles can be clustered by anatomizing the spectral graph, where the attribute profiles within a cluster have a strong connection and different clusters have a weak connection. Naturally, such algorithms have been widely applied in the field of image segmentation (Shi and Malik, 2000) neural information processing (Ng et al., 2002) biology (Zare et al., 2010) and large-scale assessment in psychology (Chen et al., 2017). However, no study has been done to investigate the performance of the SCA in CDA yet to our knowledge. And it is interesting to inspect the efficiency of the SCA for clustering examinees’ into attribute-homogeneous groups under variedly underlying processes, such as conjunctive, disjunctive, additive, and saturated model (de la Torre, 2011).

In the next section, the G-DINA model and its related reduced models will be briefly reviewed. Subsequently, the K-means and SCA algorithms are detailedly introduced in the third section. This is followed by the simulation studies comparing SCA to K-means algorithm and CDMs mentioned in the second section are conducted in section “Simulation Studies,” and the section “Analysis of Mixed Number Subtraction Data” concerns a real data study to examine the performance of the SCA. Finally, Summary and discussions are given in the final section.

## Cognitive Diagnostic Models

First, some basic concepts and terms used in CDA are introduced. Consider *J* binary item response variables for each of the *I* examinees. Let *X*_*ij*_ represent the response of examinee *i* to item *j*, where *i* = 1, 2, …, *I* and *j* = 1, 2, …, *J*. Let α_*i*_ = (α_*i*1_,α_*i*2_,…,α_*iK*_) denote the attribute profile of examinee *i*, where *K* is the number of attributes measured by the test. A value of α_*ik*_ = 1 indicates the *i*th examinee masters the *k*th attribute and α_*ik*_ = 0 otherwise. Let **q**_*j*_ = (*q*_*j*1_,*q*_*j*2_,…,*q*_*jK*_) represent the *j*th row of the **Q**-matrix that describes the relationship between items and attributes (Tatsuoka, 1995). **Q** is a *J* × *K* matrix with the entry *q*_*jk*_ = 1 indicating that item *j* requires attribute *k*, and *q*_*jk*_ = 0 otherwise.

### The G-DINA Model

The G-DINA model is able to distinguish $2^{K_{j}^{*}}$ latent classes, where $K_{j}^{*}$ is the number of required attributes for *j*th item, and $K_{j}^{*}=\sum_{k=1}^{K}q_{jk}$. For simplicity, the first $K_{j}^{*}$ attributes are treated as the required attributes for *j*th item, and $\alpha_{lj}^{*}$ is the reduced attribute vector corresponding to the columns of the required attributes with $l=1,\ldots ,2^{K_{j}^{*}}$. The probability of a correct response to *j*th item by examinees with attribute profile $\alpha_{lj}^{*}$ can be denoted by $P\left(X_{j}=1|\alpha_{lj}^{*}\right)=P\left(\alpha_{lj}^{*}\right)$. Then, the item response function (IRF) of the G-DINA model is as follow:

(1) $$\begin{array}{c} f\left[P\left(\alpha_{lj}^{*}\right)\right]=\gamma_{j0}+\sum_{k=1}^{K_{j}^{*}}\gamma_{jk}\alpha_{lk}\\ \;+\sum_{k^{\prime }=k+1}^{K_{j}^{*}}\sum_{k=1}^{K_{j}^{*}-1}\gamma_{jkk^{\prime }}\alpha_{lk}\alpha_{lk^{\prime }}+\ldots +\gamma_{j12\ldots K_{j}^{*}}\prod_{k=1}^{K_{j}^{*}}\alpha_{lk}0 \end{array}$$

where $f\left[P\left(\alpha_{lj}^{*}\right)\right]$ represents $P\left(\alpha_{lj}^{*}\right)$, $\log\left[P\left(\alpha_{lj}^{*}\right)\right]$ and $\log it\left[P\left(\alpha_{lj}^{*}\right)\right]$ in the identity, log and logit links, respectively. Moreover, γ_*j*0_ is the intercept for *j*th item, γ_*jk*_ is the main effect due to α_*k*_, γ_*jkk’*_ is the interaction effect due to α_*k*_ and α_*k’*_, and $\gamma_{j12\ldots }K_{j}^{*}$ is the interaction effect due to $\alpha_{1},\ldots ,\alpha_{K_{j}^{*}}$. For more details about the G-DINA model, please refer to de la Torre (2011).

### Related Reduced Models

It’s conspicuous that the G-DINA model is a saturated model which can easily change into several popular reduced CDMs, including the DINA model, the DINO model, the *additive* CDM (*A*-CDM), etc. Note the symbol γ is used as item parameters across all these models in this paper. So, if we set all terms in the G-DINA model in identity link except γ_*j*0_ and $\gamma_{j12\ldots }K_{j}^{*}$ to zero, the DINA model will be obtained, that is,

(2) $$P\left(\alpha_{lj}^{*}\right)=\gamma_{j0}+\gamma_{j12\ldots K_{j}^{*}}\prod_{k=1}^{K_{j}^{*}}\alpha_{lk}$$

If the intercept and main effect terms are remained with the follwing constraints: $\gamma_{jk}=-\gamma_{jk^{\prime }k^{\prime \prime }}=\ldots ={\left(-1\right)}^{K_{j}^{*}+1}\gamma_{j12\ldots K_{j}^{*}}$, for $k=1,\ldots ,K_{j}^{*}$, $k^{\prime }=1,\ldots ,K_{j}^{*}-1$, and $k^{\prime \prime }>k^{\prime },\ldots ,K_{j}^{*}$. The DINO model can be given by

(3) $$P\left(\alpha_{lj}^{*}\right)=\gamma_{j0}+\gamma_{jk}\alpha_{lk}$$

By setting all interactions to zero in the identity-link G-DINA model, the *A*-CDM can be formulated as

(4) $$P\left(\alpha_{lj}^{*}\right)=\gamma_{j0}+\sum_{k=1}^{K_{j}^{*}}\gamma_{jk}\alpha_{lk}$$

Clearly, quite a few parameters of items and examinees require estimating in the saturated model and its related reduced CDMs. More often than not, one can use either marginalized maximum likelihood estimation (MMLE) or Bayesian approach with the Markov Chain Monte Carlo (MCMC) method to achieve parameter estimation.

## Clustering Methods for Cognitive Diagnosis

### K-Means Method for Cognitive Diagnosis

K-means cluster analysis is widely used as the process of grouping a set of subjects into clusters so that subjects within a cluster have similarity in comparison to one another, but are dissimilar to subjects in other clusters. This approach finds the *k* centroid, where the coordinate of each centroid is the means of the coordinate of the subjects in the cluster and assigns every subject to the nearest centroid. Chiu et al. (2009) have made the best of K-means method in CDA already, and showed its effectiveness empirically for placing examinees in homogeneous groups. The algorithm in CDA can be summarized as follows (Please refer to Chiu et al.’s paper for details).

Step 1: Select *M* initial *K*-dimensional cluster centroids.
Step 2: Assign data points to clusters that have the closest centroid.
Step 3:When all data points have been assigned, update the positions of the *M* centroids.
Step 4: Repeat Steps 2 and 3 until the centroids no longer change.

Although K-means is a more than effective method for clustering, the starting values exercises a large impact on the classified performance for this method. Having poor starting values can result in converging to local optima (Steinley, 2003). So, many methods of choosing starting values for the K-means method have been proposed. Chiu et al. (2009) have investigated the performance of K-means method in CDA with two different kinds of starting values, called best and Ward’s cases, respectively, which provided decent clustering results, and they should be considered in this study. Additionally, the K-means with random starting values will be deemed as the baseline to compare the classification performance to other two starting values. The introduction of starting values presents in section “The Selection of Starting Values” subsequently.

### Spectral Clustering for Cognitive Diagnosis

As mentioned above, the SCA method was used in many research fields. For psychological assessment study, Chen et al. (2017) applied SCA to the context of exploratory item classification. Through constructing a graph of items, the similar items could be classified together and the dissimilar ones can be extracted based on the graphical structure. Intuitively, it is straightforward to wonder how the SCA performs on person classification in CDA. The SCA can be available in CDA context for the following reasons: (a) SCA creates a graph of examinees based on the similarity measurement of each pair of examinees, where examinees who possess the same attribute profiles tend to be connected. (b) Cai et al. (2005) wrote that “*The spectral clustering usually clusters the data points using the top eigenvectors of graph Laplacian, which is defined on the affinity matrix of data points*”. In order to construct the affinity matrix for binary response data in CDA, the *Gaussian kernel* function can be applied according to Ng et al. (2002). Then, one can use SCA to classify examinees. (c) both SCA and K-means method belong to clustering approach, and K-means is a component of the SCA method (Chen et al., 2017) which means both methods have the same parts of processing data to get clustering results. Chiu et al. (2009) had proved the feasibility of K-means in the aspect of classifying examinees into groups with same attribute profiles. So, the SCA should have a good chance of success in characterizing the same structure (i.e. attribute profiles) among examinees. We focus on the specific illustration and detail the core procedures on how to implement the SCA in CDA [for more details about the SCA, please refer to Von Luxburg (2007) and Chen et al. (2017)], now that the key point of this paper is not to introduce the SCA itself. One can easily operate this algorithm in CDA with following steps:

Step 1: Using response data to construct similarity matrix **S**, which is a *I* × *I* square matrix with element,
  (5) $$\begin{array}{c} S\left({\text{X}}_{i},{\text{X}}_{i^{\prime }}\right)=\exp\left(-{∥{\text{X}}_{i}-{\text{X}}_{i^{\prime }}∥}^{2}/2\sigma^{2}\right),\\ \;i,i^{\prime }\in \left\{1,2,\ldots ,I\right\}, \end{array}$$
  where **X**_*i*_ and ${\text{X}}_{i^{\prime }}$ are *i*th and *i’*th examinee’s response vectors. Generally speaking, one may take σ^2^ = 1 as assumption under standard normal distribution, and Eq. 5 can be considered as *Gaussian Kernel*. The SCA divided examinees into diverse clusters so that examinees in the same cluster tend to be similar, which means $S\left({\text{X}}_{i},{\text{X}}_{i^{\prime }}\right)$ value tends to be large if examinees *i* and *i’* belong to the same cluster. Meanwhile, those who are classified into different clusters tend to be differ from each other so as to the values become small.
Step 2: Construct a diagonal matrix **D**_*I*×*I*_ and compute the normalized Laplacian matrix **L**_*I*×*I*_ as follows:
  (6) $$D_{ii}=\sum_{i^{\prime }=1}^{I}S_{ii^{\prime }}$$
  and
  (7) $${\text{L}}_{I\times I}=\text{I}-{\text{D}}^{-\frac{1}{2}}{\text{SD}}^{-\frac{1}{2}}$$
  where **I** is a *I*×*I* unit matrix.
Step 3: Compute the first *M* eigenvectors **u**_1_,**u**_2_,…,**u**_*M*_ of **L**_*I*×*I*_.
Step 4: Let **U**_*I*×*M*_ be the matrix containing the vectors **u**_1_,**u**_2_,…,**u**_*M*_ as columns.
Step 5: Derived the matrix **T**_*I*×*M*_ from **U**_*I*×*M*_ by normalizing the rows to norm 1, which is set $t_{im}=u_{im}/{\left(\sum_{m}u_{im}^{2}\right)}^{\frac{1}{2}}$.
Step 6: For *i* = 1,…, *I*, let **Z**_*i*_ = (*Z*_*i*1_,*Z*_*i*2_,…,*Z*_*iM*_) be the vector corresponding to the *i*th row of **T**_*I*×*M*_.
Step 7: Cluster the points {**Z**_*i*_, *i* = 1, 2, …, *I*} with the K-means algorithm into *M* clusters.
Step 8: Assign the original points **X***_*i*_* to cluster *j* if and only if the points **Z***_*i*_* was assigned to cluster *j*.

According to these eight steps, examinees can be grouped into different clusters representing different attribute profiles. Currently, the R package “Kernlab” (Karatzoglou et al., 2004) can implement SCA availably.

### The Selection of Starting Values

#### K-Means With Best Starting Values

In order to group examinees into the correct attribute profiles, Chiu et al. (2009) introduced the sum-score statistic, which was also used in Henson et al. (2007). For the *i*th examinee, the sum-score on attribute *k* can be defined as:

(8) $$W_{ik}=\sum_{j}^{J}X_{ij}q_{jk}$$

Thus, **W**_*i*_ = (*W*_*i*1_,*W*_*i*2_,…*W*_*iK*_) is the corresponding vector of *K* sum-scores. The matrix **W**_*I*×*K*_ is then taken as the input of cluster analysis, with a fixed *M* clusters in CDA. Based on **W**_*I*×*K*_ matrix, the K-means method assigns data point **W**_*i*_ to the *m*th cluster using Euclidean distance if

(9) $$m=\arg\min_{u\in \left\{1,\ldots ,M\right\}}{∥{\text{W}}_{i}-{\hat{\text{c}}}_{u}∥}^{2}$$

Where ${\hat{\text{c}}}_{u}$ is the provisional centroids of the *u*th cluster during the iterative steps, and is calculated by averaging the observations in the cluster.

A key point of using K-means method is the selection of initial values. Let α_*m*_ = (α_*m*1_,α_*m*2_,…α_*mK*_)′ be the *unique* attribute profile in the universal set of attribute profiles, where *m* = 1, 2, …, *M* and *M* = 2^*K*^. For example, only four attribute profiles exist when *K* = 2, and they are α_1_ = (0, 0), α_2_ = (0, 1), α_3_ = (1, 0), and α_4_ = (1, 1), respectively. Then, the initial value matrix (denoted as **W**_*M*×*K*_) in the ‘best’ scenario can be calculated as follow:

(10) $${\text{W}}_{M\times K}={\text{P}}_{M\times J}{\text{Q}}_{J\times K}$$

where **P**_*M*×*J*_ is the expected response matrix with entry *p*_*mj*_ indicating that the probability of *m*th attribute profile correctly answering *j*th item. For instance, *p*_*mj*_ should be calculated according to Eq. 2 if the DINA model is selected (Chiu et al., 2009). Note that *p*_*mj*_ is used only as an ideal state for comparison in simulation study. When implementing K-means in practice, we have no idea about *p*_*mj*_ actually, thus other starting values, i.e. random and Ward’s, will be selected.

#### Clustering With Ward’s Starting Values

Ward’s method is a general agglomerative hierarchical clustering approach originally presented by Ward (1963). The criterion of this manner is to minimize the total within-cluster variance. To implement this method, at each step find the pair of clusters that leads to minimum increase in total within-cluster variance after merging. This increase is a weighted squared distance between cluster centroids, and can be represented as the sum of square errors (SSE) statistic. Suppose that cluster *p* and *q* are next to be merged. Then, the SSE for the *p*th cluster is computed as follow:

(11) $$SSE_{p}=\sum_{i=1}^{I_{p}}{\left({\text{Y}}_{pi}-{\bar{\text{Y}}}_{p}\right)}^{\prime }\left({\text{Y}}_{pi}-{\bar{\text{Y}}}_{p}\right)$$

where *I*_*p*_ and *I*_*q*_ represent the number of data in clusters *p* and *q*, respectively. **Y**_*pi*_ is *i*th data point in cluster *p*, and ${\bar{\text{Y}}}_{p}$ is the centroid of cluster *p*. Using the Eq. 11, the SSE for the *q*th cluster can be got. So, the *p*th and the *q*th clusters are merged into a new cluster if

(12) $$SSE_{pq}-\left(SSE_{p}+SSE_{q}\right)=\frac{I_{p}I_{q}}{I_{p}+I_{q}}{\left({\bar{\text{Y}}}_{p}-{\bar{\text{Y}}}_{q}\right)}^{\prime }\left({\bar{\text{Y}}}_{p}-{\bar{\text{Y}}}_{q}\right)$$

is the minimum among all pairs, where *SSE*_*pq*_ is the combined SSE for cluster *p* and *q*.

Subsequently, the initial values are determined according to the result of Ward’s method in the ‘Ward’s starting values’ scenario.

#### Clustering With Random Starting Values

The simplest method of choosing initial values is to utilize the random procedure. That means *M* data points may be selected randomly from the data set, and be treated as the *M* cluster centroid. Now that there is no prior knowledge guiding the way to choose the starting values in ‘random’ scenario, the randomness exerts a significant influence on the performance of this method. Then, with random starting values, the K-means and SCA can be considered as the baseline for the study.

Note that the ‘best’ starting value is used in K-means method but excluded in the SCA because the dimensionality of the matrix **W**_*M*×*K*_ is different from the matrix **Z**_*I*×*M*_. However, other two starting values can be both applied for SCA and K-means. The SCA and K-means are comparable as the following reasons: On the one hand, Chen et al. (2017) indicated that the K-means method is a component of the SCA algorithm. Meanwhile, the original materials used by both SCA and K-means method are raw response data actually. Only difference between these two methods is the mean to tackle raw response data. For the K-means method, in order to get the consistency theory, raw data was reconstructed through **W**_*I*×*K*_ = **X**_*I*×*J*_**Q**_*J*×*K*_, and **W**_*I*×*K*_ matrix was used as input. On the other hand, according to the SCA, raw response data was reconstructed as **Z**_*I*×*M*_ matrix through Steps 1 to 6 described in section “Spectral Clustering for Cognitive Diagnosis.” And then, **Z**_*I*×*M*_ matrix was treated as input in K-means method. Based on these evidences, clustering results from SCA are comparable with those from K-means method in essence.

## Simulation Studies

The first goal of simulation studies is to investigate the effectiveness of clustering using the SCA in CDA, and compare SCA with K-means method in the aspect of classification accuracy further. These two methods pertain to clustering approach, and the last step of SCA needs to call K-means to accomplish clustering, which means both methods have the same parts of processing data to get clustering results. However, hamming distance is excluded in this paper because this method requires prior knowledge of cognitive processes to obtain the ideal response patterns. Then, measures of distance between observed response patterns and ideal response patterns can be calculated. It indicates that hamming distance method need to know the mechanism between attributes in advance (Chiu and Douglas, 2013). The SCA and K-means methods are unstinted in this constraint, clustering examinees according to their responses only.

Besides, it is not clear that the performance of K-means method is under some particular underlying processes (e.g. additive and saturated scenarios) because there is no research to compare K-means with the *A*-CDM and G-DINA model. So, the second goal is to examine the performances of the SCA and K-means methods in processing various response data sets generated by different CDMs, including the G-DINA, DINA, DINO, and *A*-CDM.

### Simulation Design

To evaluate the performance of the SCA in clustering examinees, five factors were manipulated: the number of examinees *I* was set to 100 or 500; The number of attributes *K* equaled 3, 4 or 5; The item quality was defined by two parameters, which were denoted as 1 − *P*(1) and *P*(1). Items with 1 − *P*(1), *P*(1) ∈ *U*(0.05, 0.15) were labeled high quality, and items with 1 − *P*(1), *P*(1) ∈ *U*(0.25, 0.35) were low quality (Ma et al., 2016); Generating models were G-DINA, DINA, DINO, and *A*-CDM model, respectively; Test length *J* = 5, 10, or 20. The generating rules of Q-matrix were as follows: (a) ensure that there were items at least require one attribute in Q-matrix. (b) the remaining items were selected from all 2^*K*^ − 1 items randomly to satisfy the predetermined test length. For each condition, 100 replications were used.

The true attribute profiles α were linked to an underlying multivariate normal distribution (Chiu et al., 2009) **θ**_*i*_∼*MVN*(**0**_*K*_, Σ), where the covariance matrix Σ is

$$\left(\begin{array}{ccc} 1\; & & \rho \\ & \ddots \; & \\ \rho \; & & 1 \end{array}\right)$$

Where ρ was set to 0.5, representing medium correlation between attributes. Let **θ**_*i*_ = (θ_*i*1_,…θ_*iK*_)′ express the latent continuous ability for examinee *i*, the attribute profile α_*i*_ = (α_*i*1_,…,α_*iK*_)′ was calculated by

(13) $$\alpha_{ik}=\left\{ \begin{array}{cc} 1\; & if\theta_{ik}\geq \Phi^{-1}\left(\frac{k}{K+1}\right),\\ 0\; & otherwise. \end{array}\right.$$

### Evaluation Criteria

To evaluate the performance of classifications in CDA, attribute correct classification rate (ACCR) and pattern correct classification rate (PCCR) are commonly used as the indicators. Nevertheless, they become available when examinees are classified into labeled sets, which is not the case with cluster analysis, for the reason that they manifest the consistency between the true and estimated attribute profiles. Only when the estimates of examinees’ attribute profiles cognized can these indices be calculated. Obviously, it is not an issue when researchers use CDMs to analyze response data. However, the cluster analysis classifies examinees into attribute-homogeneous groups, but it cannot provide information about the estimates of examinees’ attribute profiles (i.e. labeling problem). So, ACCR and PCCR indices cannot be calculated in this case. Therefore, two indices which were applied in Chiu et al. (2009) paper were also used in this study. One was an indicator of agreement between partitions, called the Adjusted Rand Index (ARI), and the other was denoted as ω assessing the within-cluster homogeneity.

The ARI was modified from Rand index, and was originally proposed by Hubert and Arabie (1985). Given a set of *I* examinees *S* = {*O*_1_, …, *O*_*I*_}, suppose that *U* = {*u*_1_, …, *u*_*R*_} and *V* = {*v*_1_, …, *v*_*G*_} represent two different partitions of the examinees in *S*. Supposed that *U* is the external criterion, i.e. true attribute profile in CDA, and *V* is a clustering result. The ARI assumes the generalized hypergeometric distribution as the model of randomness, i.e. the *U* and *V* partitions are picked at random such that the number of examinees in the clusters are fixed. Let *I*_*rg*_ be the number of examinees that are in both classes *u*_*r*_ and *v*_*g*_, where *r* = 1, 2,…, *R*, and *g* = 1, 2,…, *G*. Let *I*_*r*•_ and *I*_•*g*_ be the number of examinees in class *u*_*r*_ and *v*_*g*_, respectively. Then, the ARI can be shown as follows:

(14) $$ARI=\frac{\sum_{r,g}C_{I_{rg}}^{2}-\sum_{r}C_{I_{r•}}^{2}\sum_{g}C_{I_{•g}}^{2}/C_{I}^{2}}{\frac{1}{2}\left[\sum_{r}C_{I_{r•}}^{2}+\sum_{g}C_{I_{•g}}^{2}\right]-\sum_{r}C_{I_{r•}}^{2}\sum_{g}C_{I_{•g}}^{2}/C_{I}^{2}}$$

which is limited between 0 and 1. The larger the ARI is, the higher agreement between partitions is. In Eq. 14, a binomial coefficient $C_{\left(•\right)}^{2}$ is defined as 0 when the number of classified objects is 0 or 1.

In CDA, the index ω which can be used to evaluate the within-cluster homogeneity with respect to the true attribute profiles measures how similar examinees from the same cluster are to one another, and sums this over the clusters (Chiu et al., 2009). The formula for ω is given by

(15) $$\omega =1-\frac{\sum_{i=2}^{I}\sum_{i^{\prime }=1}^{i}\sum_{k=1}^{K}\left|\alpha_{ik}-\alpha_{i^{\prime }k}\right|{\text{I}}_{\left[{\hat{c}}_{i}={\hat{c}}_{i^{\prime }}\right]}}{\sum_{i=2}^{I}\sum_{i^{\prime }=1}^{i}K\times I\left[{\hat{c}}_{i}={\hat{c}}_{i^{\prime }}\right]}$$

where ${\hat{c}}_{i}$ represents the classified result for the *i*th examinee, and *I*_[*č_i_*=*č_i_*′]_ is the indicator function reflecting whether or not examinees *i* and *i’* are classified into same cluster. This index is also bounded between 0 and 1, and it equals 1 if true attribute profiles are the same for all pairs of examinees clustered together.

### Results

**FIGURE 1:**
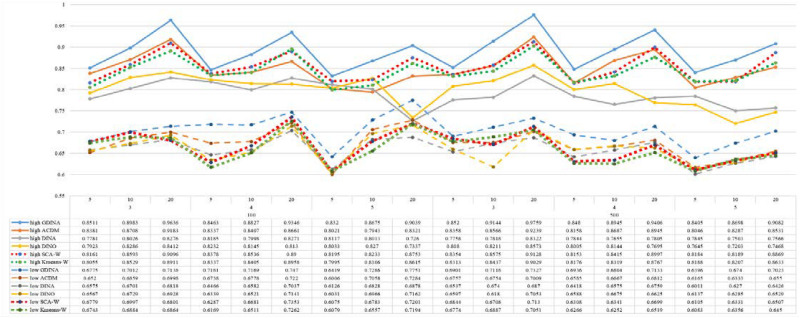
Mean values of ARI by SCA, K-means, and fitted models; True model = G-DINA.

**FIGURE 2:**
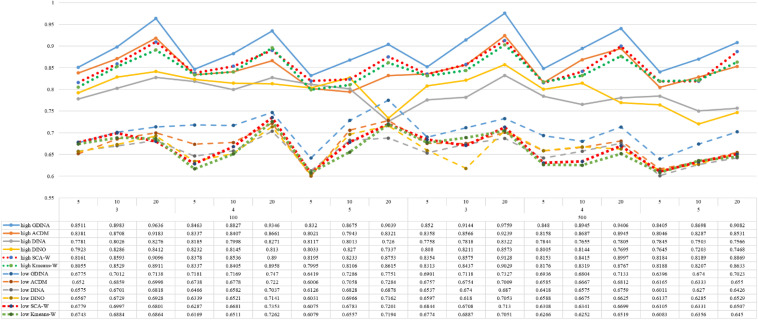
Mean values of ω by SCA, K-means, and fitted models; True model = G-DINA.

**FIGURE 3:**
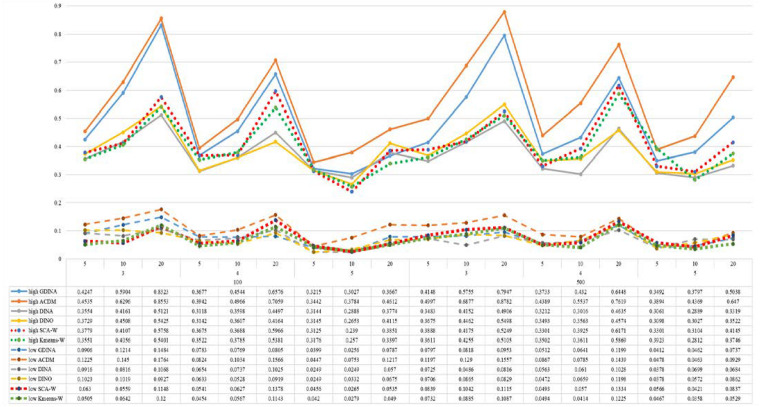
Mean values of ARI by SCA, K-means, and fitted models; True model = *A*-CDM.

**FIGURE 4:**
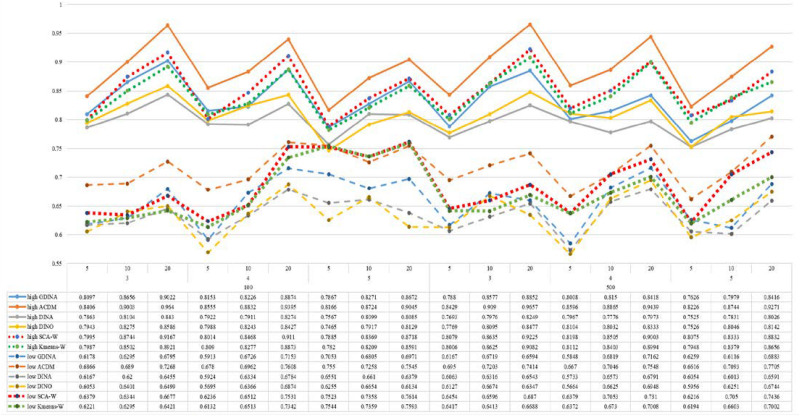
Mean values of ω by SCA, K-means, and fitted models; True model = *A*-CDM.

**FIGURE 5:**
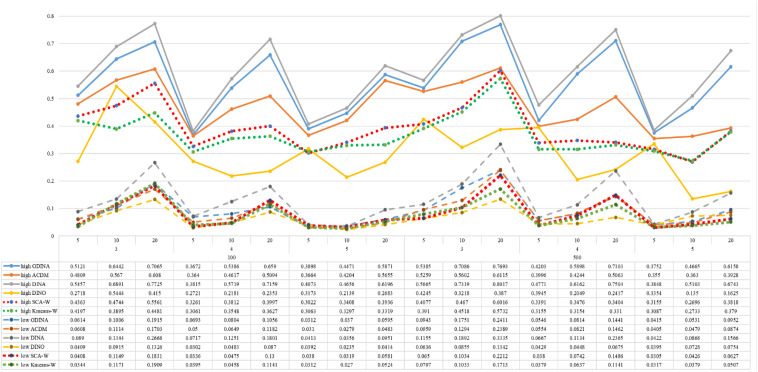
Mean values of ARI by SCA, K-means, and fitted models; True model = DINA.

**FIGURE 6:**
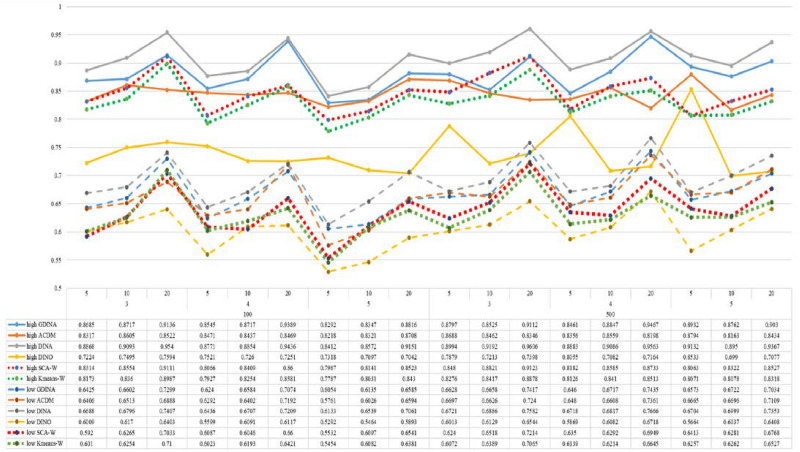
Mean values of ω by SCA, K-means, and fitted models; True model = DINA.

**FIGURE 7:**
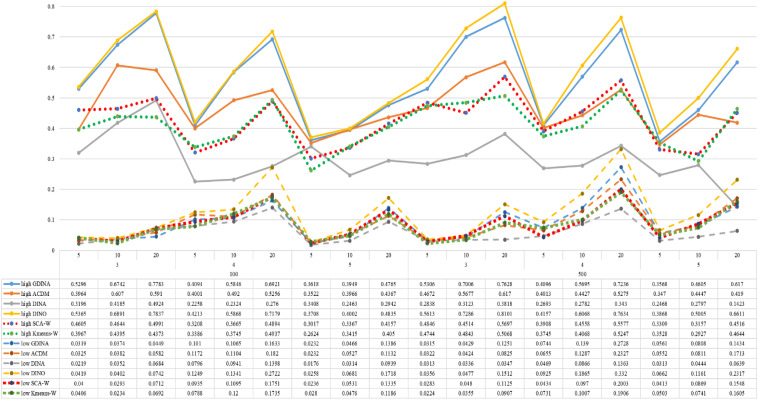
Mean values of ARI by SCA, K-means, and fitted models; True model = DINO.

**FIGURE 8:**
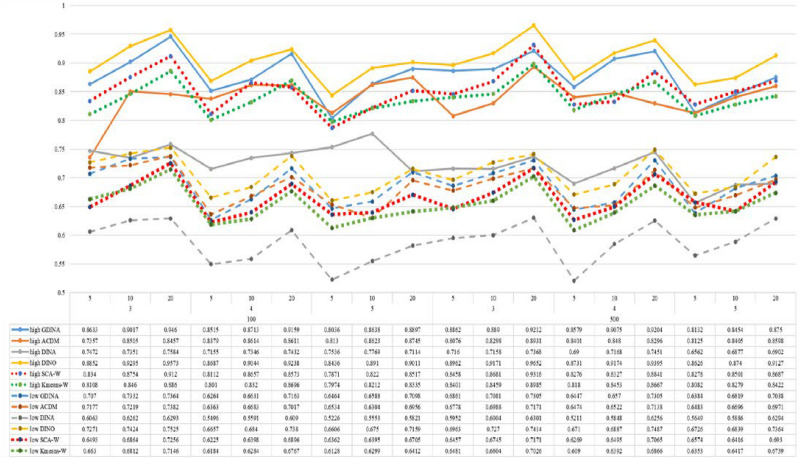
Mean values of ω by SCA, K-means, and fitted models; True model = DINO.

Figures 1–8 totally demonstrate the means of ARI and ω for SCA, K-means, G-DINA model and its related reduced CDMs over 100 replications for each condition. Classification results of the true model are definitely the best, which provides the upper limit of comparison across all conditions. Oppositely, the random case just provides the lower limit of comparison to other settings, and it has indicated the worst performance among all methods based on simulation results. Although the “best” scenarios are treated as the best possible case for K-means to cluster response data, it has to use CDMs to get the expected response *p*_*mj*_ in advance, then **W** can be calculated. In this sense it is not indeed a nonparametric method. So, we mainly compare the performances of Ward’s linkage for two clustering methods against the ones of other fitted CDMs in the following. The results of SCA with random, K-means with random and K-means with best do not present here.

According to all results, the ARI and ω values are comparable between SCA and other methods (K-means and fitted CDMs) on the whole. In each Figure, the lines are clearly divided into two parts on account of item quality. The top half part presents high quality while the bottom half part presents low quality conditions. These results fully reflect the item quality, with a significant influence on accuracy of classification. Take Figures 1, 2 as an example, ARI values are all above 0.3, and ω values are all larger than 0.81 under the SCA with high quality. However, the lowest values of ARI and ω are 0.0284 and 0.6075, respectively, with low quality. Figures 3–8 show the same results under different generating CDMs. It is noted that this deterioration is not unique for the SCA, moreover, the K-means and CDMs also have the same tendency. It demonstrates that item quality not only has a prominent influence on the performance of CDMs, but also has a dramatical effect on clustering methods. So, some important attentions should be paid to item quality in order to promote the classification accuracy in CDA regardless of the particular classification methods. As for two clustering methods, SCA can obtain higher ARI and ω values, representing more accurate clustering in most conditions, which can be concluded from that the red dot line (the legend denoted as SCA-W) is mostly above the green dot line (the legend denoted as Kmeans-W) in each parts.

For sample size, the impact of this factor on classification accuracy of these approaches is almost the same when other factors (e.g. attribute number, test length, item quality, and true models) are fixed, which means the clustering performance of SCA is comparable to K-means and other fitted CDMs. As can be seen those from eight figures, the ARI and ω values, soaring as the sample size, become large (from 100 to 500) on the whole. Since the relative advantage of cluster analysis applicated in small sample size, the main outcomes had been described under 100 sample size conditions (the left half part in each figure). Note that the similar results are presented in 500 sample size condition. When the G-DINA is the true model, the ARI and ω values of SCA are higher than those from K-means, DINA and DINO models (the red dot line is above) except that the ARIs in the conditions *K* = 3 and item quality is high, and *K* = 4 and item quality is low, respectively. This indicates SCA can be applied to most tests where there are a saturated underlying processes between attributes. As for *A*-CDM is the true model, we can see that SCA performs better than K-means, DINA and DINO models when item quality is high (except *K* = 5). Futhermore, SCA performs similarly as others in terms of ARIs (except *K* = 3 and *J* = 5 or 10), but ω values are consistently higher than other methods when item quality is low, which demonstrates stronger within-cluster homogeneity. This suggests SCA can also obtain decent classification accuracy when the cognitive mechanism is additive between attributes. Considering the true model is DINA model, the ARIs from SCA are almost higher than those from K-means and DINO model. Meanwhile, the ω values from SCA are also the highest among these three methods when item quality is low, whereas the magnitudes of ωs are affected by test length when item quality is high. Specifically, ω values of SCA are higher than those from K-means, *A*-CDM and DINO when *J* = 20, and be inverse when *J* = 5 or 10. The results show that the performance of SCA is acceptable when item quality is low, or J > 20 if the underlying process is conjunctive among attributes. Providing that DINO is the true model, the ARIs from SCA are almost higher than those from K-means and DINA model. Similarly, the ω values from SCA are the highest among these three methods, especially higher than *A*-CDM when *K* = 3 and item quality is high. This implies SCA has a patchy performance when disjunctive process arose between attributes.

In addition, the number of attributes also affects the classification accuracy of SCA as same as CDMs. Generally speaking, with attribute number *K* increasing, the ARI and ω values decreases. Most results conform to this pattern as shown in Figures 1–8. However, this trend is not consistent across all conditions. For instance, in Figure 1, for condition (*I*, *J*) = (100, 5), ARI values change from 0.3554 to 0.3628 under SCA, while ARI values change from 0.3446 to 0.3754 under K-means when K grows from 3 to 4. ω values change from 0.8161 (0.8055) to 0.8378 (0.8337) under SCA (K-means). Due to the randomness of generating Q-matrix in each replication, the K-means may arise some reversal results in some conditions. So, it may infer that the combination of *q*-vectors influences the effect of attribute number on classification accuracy.

Last, test length is a widely considered factor in CDA. Many studies have discussed the influence of this factor on classification accuracy (Chen et al., 2013; Chiu et al., 2009). From the results of these simulations, as *J* increases, the classification abilities of all methods tend to improve. Considering the shortest test length condition (*J* = 5), most ω values are no less than 0.8 when item quality is high, while most ω values are no less than 0.6 when item quality is low under the SCA procedure. Definitely, the longer the test length is, the more information about the examinees it provides, and more accurate classification will be obtained. This indicates the SCA can be affected by test length just like other methods.

## Analysis of Mixed Number Subtraction Data

### Data Description

The data consist of 536 examinees’ responses to 11 items taken from the mixed number fraction subtraction. The **Q**-matrix was modified from five attributes to three attributes, and they were previously used by Henson et al. (2009). The attributes defined for this study are (1) borrowing from a whole number, (2) separating a whole number from a fraction and (3) determining a common denominator. Table 1 shows the 11 items and their required attributes. It should be pointed out that the data and the **Q**-matrix were got from R package ‘CDM’, and the item 12 was excluded from the original table as shown in Henson et al.’s paper. So, there were just 11 items in this study. Then, the SCA and K-means algorithm with Ward’s linkage, and four CDMs were applied to classify examinees into different clusters.

**TABLE 1:** Mixed number fraction subtraction and corresponding q-matrix.

Item number	Item	Q-matrix	Item number	Item	Q-matrix
1	$3+\frac{1}{2}-2+\frac{3}{2}$	1 1 0	8	$2-\frac{1}{3}$	1 0 1
2	$3-2+\frac{1}{5}$	1 0 1	9	$4+\frac{5}{7}-1+\frac{7}{4}$	1 1 1
3	$3+\frac{7}{8}-2$	1 0 1	10	$7+\frac{3}{5}-\frac{4}{5}$	1 0 0
4	$4+\frac{4}{12}-2+\frac{7}{12}$	1 0 0	11	$4+\frac{1}{10}-2+\frac{8}{10}$	1 0 0
5	$4+\frac{1}{3}-2+\frac{4}{3}$	1 1 0	13	$4+\frac{1}{3}-1+\frac{5}{3}$	1 1 0
6	$\frac{11}{8}-\frac{1}{8}$	1 1 0			

Two major criteria evaluating the classified quality were used as those in Chiu et al. (2009) study, denoted as within-cluster mean of **W** (see Eq. 10 for the definition), and square root of mean squared residual (MSR) of **W**. Specifically, the mean of **W** reflects how well-separated cluster means are, which can provide good identification of examinees’ overall patterns. And MSR of **W** shows that how homogeneous a cluster is. The MSR of **W** for cluster *m* is given by

(16) $$MSR\left(m\right)=\frac{\sum_{i}^{I_{m}}{∥{\text{W}}_{i}^{\left(m\right)}-{\bar{W}}^{\left(m\right)}∥}^{2}}{I_{m}}$$

where *I*_*m*_ is the number of examinees grouped into cluster *m*. The smaller the MSR is, the more homogeneous a cluster is.

Meanwhile, we also report the cluster size and mean of sum-score as the auxiliary indicators. The classification results from SCA, K-means, and CDMs were sorted by means of sum-score, which can be used to infer attribute profiles in practice (Chiu et al., 2009). The rationale is that one may get higher sum-score if (s)he masters more attribute in a test usually.

### Analysis and Results

The data were analyzed by all methods through the statistic **W**. We only select the Ward’s starting values due to their good performance in simulation studies. Note that the attribute profiles’ labels were not available for clustering analysis, and the results from the SCA and K-means were sorted along with the means of sum-scores in the same cluster, illustrating how one can infer the examinees’ attribute profiles. It means that the mean of sum-scores in certain cluster representing α = (0, 0, 0) is definitely the smallest among eight attribute profiles, while the mean of sum-scores is the largest for profile α = (1, 1, 1). Because of the acquirement of specific attribute profiles by using the CDMs, results are listed according to the size of attribute vectors.

When using multiple models to fit the same data, the Akaike’s information criterion (AIC; Akaike, 1974) and the Bayesian information criterion (BIC; Schwarzer, 1976) were usually adopted to determine which model can provide a better fit result. For each of these two statistics, the fitted model with a the smaller value is selected among the set of competing models. Table 2 shows the AIC and BIC for four CDMs fitting the fraction subtraction data. The AIC is the smallest under the G-DINA model, but the BIC is the smallest under A-CDM. According to previous study, if AIC and BIC contradict each other, the BIC may provide a better result for selecting model because BIC takes into account both the sample size and the number of parameters of the model (Chen et al., 2013). Based on this point, the A-CDM provides the best fit among these four CDMs.

**TABLE 2:** AIC and BIC for four CDMs fitting fraction subtraction data.

Models	AIC	BIC
G-DINA	**5341.06**	5550.98
DINA	5534.39	5658.63
DINO	5517.80	5642.04
A-CDM	5363.15	**5525.94**

*Bold values mean the best models that we need to select to fit the data.*

Due to the space limitation, only the results obtained by the best fit model, *A*-CDM, are shown in the table. As can be seen in Table 3, the *A*-CDM intensively grouped most examinees into three main clusters, and the remaining clusters only have a few examinees. In addition, the differences among W_1_ to W_3_ are comparative large under the *A*-CDM, so it is benificial to identify attribute profiles of examinees. However, large MSRs are got by using this model, which means this empirical data are not clustered closely based on examinees’ profiles, then apart cluster means may result in heterogeneous clustering.

**TABLE 3:** Classification by A-CDM.

Profile	Size	Mean W			$\sqrt{MSR\left(m\right)}$	Mean Sum-score
		W_1_	W_2_	W_3_		
(0 0 0)	127	0.93	0.51	0.66	1.35	1.07
(0 1 0)	109	1.55	1.94	1.37	1.34	2.83
(0 0 1)	6	3.41	1.33	3.00	0.75	4.00
(1 0 0)	9	4.70	3.56	1.22	1.19	6.11
(1 1 0)	40	3.62	4.55	1.45	1.35	7.33
(1 0 1)	6	2.59	2.83	3.50	1.03	8.00
(0 1 1)	44	4.37	2.14	2.93	1.60	4.80
(1 1 1)	195	7.09	4.63	3.62	1.37	9.88

In contrast, Table 4 shows that the SCA classified most data to the profiles which stand for mastering only one attribute (denoted as α^(1)^), the number of examinees is 137. The second largest cluster size is 75, and this cluster represents the profile α = (1, 1, 1). Similarly, the K-means method also classified most data to the same profiles, with the clusters α^(1)^ and α = (1, 1, 1) are both containing 100 examinees. The distances between the pairs of clusters in the SCA are larger than those in K-means method according to the values of **W**, which means that SCA can give well-separated clusters. In addition, the values of MSR under these two clustering methods are smaller than those under the *A*-CDM. Further, MSR under SCA are smaller than those under K-means, except one cluster (see the bold value on the second row). This is in accord with the results from simulation study that the SCA tends to form close and homogeneous clusters.

**TABLE 4:** Classification by SCA-Ward’s and K-means-Ward’s algorithm.

Size	Mean W			$\sqrt{MSR\left(m\right)}$	Mean sum-score
	W_1_	W_2_	W_3_		
59 (79)	0.03 (0.04)	0.41 (0.70)	0.07 (0.09)	0.67 (0.79)	0.61 (0.73)
62 (40)	0.13 (0.00)	0.24 (0.00)	1.26 (1.00)	0.75 (**0.00**)	1.63 (1.00)
71 (100)	0.18 (0.05)	1.10 (1.04)	1.82 (1.61)	0.67 (0.74)	3.10 (2.70)
137 (71)	0.88 (0.87)	1.98 (1.24)	2.28 (2.82)	1.09 (1.18)	5.14 (4.93)
58 (52)	2.24 (2.06)	3.36 (3.35)	1.60 (1.35)	1.18 (1.30)	7.21 (6.75)
32 (52)	2.78 (2.62)	3.66 (2.87)	2.94 (3.60)	0.76 (0.77)	9.38 (9.08)
42 (42)	2.40 (2.57)	3.33 (4.00)	3.86 (2.81)	0.70 (0.93)	9.60 (9.38)
75 (100)	3.00 (2.67)	4.00 (3.97)	3.96 (4.00)	0.25 (0.55)	10.93 (10.67)

*Results of K-means-Ward’s present in parentheses.*

Finally, taking the *A*-CDM as the standard, Table 5 presents the classification agreement of each two methods, including SCA, K-means, and *A*-CDM. The agreement between the *A*-CDM and SCA is slight higher than the other pairs with an ARI of 0.468 compared to an ARI of 0.443 for the agreement between the *A*-CDM and K-means. It indicates that SCA outperformed K-means for this data set.

**TABLE 5:** ARI table for ACDM, SCA and *K*-means.

	*A*-CDM	SCA-Ward’s	K-means-Ward’s
*A*-CDM	–	0.468	0.443
SCA-Ward’s		–	0.427
K-means-Ward’s			–

## Summary and Discussion

The contribution of this study is to introduce the SCA into cognitive diagnosis and compare it with the K-means method and different CDMs in terms of classification accuracy. The clustering methods are computationally efficient and effctive for data with any sample size. It’s easy and convenient to implement, and researchers only need to know the number of required attributes and their hierarchical structures. The previous study had shown that K-means has favorable performance in clustering examinees who possess the same attribute profiles (Chiu et al., 2009). In this study, we introduced the SCA for grouping examinees’ attribute profiles into specific clusters in CDA. Then, the performance of SCA on classification accuracy was investigated under different factors, and some interesting findings were made based on simulation studies.

The most important factor affecting the classification accuracy of both clustering analysis and CDMs was item quality. Generally, the higher the item quality was, the higher the classification accuracy was. This is because the randomness (i.e. guessing and slipping behaviors) in the responses will decrease with high quality leading to a more aggregated cluster for the same attribute profile of examinees. Thus, it is not difficult to distinguish the differences between clusters.

With the number of attribute increasing, the ARI and ω values decrease for all methods. We know that the total number of attribute profiles in CDA is exponential in the number of attributes, i.e. 2^*K*^ which is also the magnitude of clusters to be identified. Obviously, the difficulty of accurately identifying attribute profiles from a large space is considerable. Besides, as test length increases, the classification abilities of all methods tend to improve. This results are consistent with previous studies. We chose short test length in simulation studies because, a) if giving students an “embedded assessment” at the end of an instruction period, we must prefer short tests to save lecture time (Wang, 2013). In addition, teachers also want to get the attribute profiles of students quickly with short test. b) some diagnostic tests that are commonly used in CDA do not have too many items, especially when the number of attributes is small. Based on our simulations, the SCA can yield considerable classification accuracy when test length is 20.

Simulation results presented here showed that the true CDM is always the best one to fit data. However, the underlying processes among attributes are various in real data actually, and it is hard to define the exact relationship between them. So, the simplicity of cluster analysis is an attractive selection without regard to specify the underlying processes in advance. As mentioned in section “Spectral Clustering for Cognitive Diagnosis,” the SCA could simply implemented via the R package called ‘Kernlab’, which means it is very easy to master by teachers and practitioners. In this study, we investigated the performance of SCA under four specifical processes (saturated, additive, conjunctive and disjunctive) and compared it with other approaches. Overall, the SCA performed comparably to fitted CDMs, and it was basically superior to K-means method. Particularly, the ω values from SCA were highest when the true model was A-CDM (excluded the true model). The strength of cluster analysis was the application in small sample size, so we mainly focused on this point in this study. When the sample size was small, the effectiveness of SCA varied depending on the mechanism of attributes according to simulation results. So, integrating the role of generating CDMs and sample size, our usage recommendation is that the SCA is suitable for analyzing data in regard to saturated and additive underlying processes while it has slightly worse efficiency in conjunctive and disjunctive scenarios. With the sample size increased, it should be pointed out that the classification accuracy became better for all these approaches and the differences in classification accuracy between clustering analysis and CDMs were shrinked.

The ARIs are generally low for some conditions in this paper. These three setting factors (i.e. item quality, test length, and generating model) in this research are different from Chiu et al. (2009) study. As for reason, we can see that these three factors have significant effects on classification accuracy based on our simulation studies. So, it is not strange that the ARIs are lower than those of conclusions in Chiu et al.’s study. In point of fact, the ARIs are not very low when item quality is high in this study.

Just like K-means, the SCA also suffers from the *labeling problem*, and has difficulty in matching each cluster to a certain attribute profile. This is a major issue of clustering analysis for CDA. However, perhaps one can draw on the teachers’ experience to help to determine the students’ attribute profiles in the classroom. This issue will be one of our future directions.

Several directions for research can be identified. First, the hierarchy of attributes refers to structurally independent in this research, which means there is no prerequisite in every required attributes. So, the correlation exists between attributes is plausible in this case. However, there are other different structures among the attributes, such as linear, convergent, divergent, and unstructured hierarchical structures (Leighton et al., 2004). The hierarchy generally defines the educational and psychological ordering among the attributes required to solve a test problem, so it is reasonable to infer the attribute structures often exists in the test (Kim, 2001). Although the performance of SCA in one of the structures has been examined in this study, it can not directly generalize to other cases without investigation. So, the effect of different attributes structures need further studies.

Second, the fully connected graph, Gaussian Kernel (Eq. 5), was used to construct similarity matrix **S** in this study. However, there are different similarity graphs in the SCA, such as the *epsilon*-neighborhood graph and *k*-nearest neighbor graphs. Besides, two major methods, the unnormalized and the normalized spectral clustering, can be used to calculate Laplacian matrix. The current paper focused only on the normalized case. In the future, other similarity graphs and unnormalized spectral clustering method should be considered in the SCA to investigate the classification ability for the CDA.

Third, as an initial research to propose the SCA into CDA, the current study only investigated the SCA’s performance for the dichotomous item responses. However, recent study proposed a general polytomous cognitive diagnosis model for a special type of graded responses to deal with non-dichotomous item responses (Ma and de la Torre, 2016). So, it is necessary to develop the clustering analysis to cope with the cognitive diagnostic test with both dichotomous and polytomous items. Thus, it may be reasonable to measure the similarity by methods based on rank correlation, such as in Chen et al. (2017). It is interesting to investigate how well the SCA performs for the graded responses.

## Data Availability Statement

The datasets generated for this study are available on request to the first corresponding author.

## Author Contributions

LG proposed the idea of the manuscript, wrote and revised the manuscript. JY wrote the simulation study code and revised the manuscript. NS organized and proofread the manuscript.

## Conflict of Interest

The authors declare that the research was conducted in the absence of any commercial or financial relationships that could be construed as a potential conflict of interest.
