# Corrigendum: Spectral Clustering Algorithm for Cognitive Diagnostic Assessment

**DOI:** 10.3389/fpsyg.2021.706512

**Published:** 2021-06-17

**Authors:** Lei Guo, Jing Yang, Naiqing Song

**Affiliations:** ^1^Faculty of Psychology, Southwest University, Chongqing, China; ^2^Southwest University Branch, Collaborative Innovation Center of Assessment Toward Basic Education Quality, Chongqing, China; ^3^School of Mathematics and Statistics, Northeast Normal University, Changchun, China; ^4^Basic Education Research Center, Southwest University, Chongqing, China; ^5^Urban and Rural Education Research Center, Southwest University, Chongqing, China

**Keywords:** cognitive diagnostic assessment, spectral clustering, K-means, G-DINA model, classification accuracy

**Funding**

This research was supported by the National Natural Science Foundation of China (31900793), the Research Program Funds of the Collaborative Innovation Center of Assessment toward Basic Education Quality at Beijing Normal University (2019-06-023-BZPK01 and 2018-06-002-BZPK01), and the Philosophy Society of the Ministry of Education (11jhq001).

The authors apologize for this error and state that this does not change the scientific conclusions of the article in any way. The original article has been updated.

